# Photomechanical Polymer Nanocomposites for Drug Delivery Devices

**DOI:** 10.3390/molecules26175376

**Published:** 2021-09-04

**Authors:** Jonathan David López-Lugo, Reinher Pimentel-Domínguez, Jorge Alejandro Benítez-Martínez, Juan Hernández-Cordero, Juan Rodrigo Vélez-Cordero, Francisco Manuel Sánchez-Arévalo

**Affiliations:** 1 Instituto de Investigaciones en Materiales, Universidad Nacional Autónoma de México, Apdo. Postal 70-360, Cd. Universitaria, México 04510, Mexico; jonathandlpzlgo@gmail.com (J.D.L.-L.); postdata.reinher@gmail.com (R.P.-D.); jbenitez1393@gmail.com (J.A.B.-M.); jhcordero@iim.unam.mx (J.H.-C.); 2 Cátedras CONACyT-Instituto de Física, Universidad Autónoma de San Luis Potosí, San Luis Potosí 78290, Mexico; jrvelez@ifisica.uaslp.mx

**Keywords:** drug delivery system, drug delivery devices, photomechanical nanocomposites, polydimethylsiloxane, carbon nanoparticles

## Abstract

We demonstrate a novel structure based on smart carbon nanocomposites intended for fabricating laser-triggered drug delivery devices (DDDs). The performance of the devices relies on nanocomposites’ photothermal effects that are based on polydimethylsiloxane (PDMS) with carbon nanoparticles (CNPs). Upon evaluating the main features of the nanocomposites through physicochemical and photomechanical characterizations, we identified the main photomechanical features to be considered for selecting a nanocomposite for the DDDs. The capabilities of the PDMS/CNPs prototypes for drug delivery were tested using rhodamine-B (Rh-B) as a marker solution, allowing for visualizing and quantifying the release of the marker contained within the device. Our results showed that the DDDs readily expel the Rh-B from the reservoir upon laser irradiation and the amount of released Rh-B depends on the exposure time. Additionally, we identified two main Rh-B release mechanisms, the first one is based on the device elastic deformation and the second one is based on bubble generation and its expansion into the device. Both mechanisms were further elucidated through numerical simulations and compared with the experimental results. These promising results demonstrate that an inexpensive nanocomposite such as PDMS/CNPs can serve as a foundation for novel DDDs with spatial and temporal release control through laser irradiation.

## 1. Introduction

Long-term treatments for chronic diseases generally rely on the adherence to a prescribed medicine intake. Several chronic diseases such as diabetes, cancer, asthma, or Parkinson’s require frequent drug administration [1]. Some drug therapies also rely on sustaining a minimal concentration of medication in the bloodstream, and they further require various doses at regular intervals of time [2]. Typically, the doses are administered orally through pills or intravenously using multiple injections, which may be inconvenient for some patients [3,4]. Controlling the spatial and temporal distribution of specific drugs in the human body still remains as an important challenge that has led to the emergence of drug delivery systems (DDS).

DDS can include devices, formulations, and technologies that allow for the release of a sustained amount of a drug over time, in a specific site within the human body, to safely and efficiently achieve a therapeutic effect [5,6,7]. DDS may include reservoirs, pumps [8], implantable biosensors [9], catheters, microfluidic devices, controlled-release microchips, molecular imprinted polymers, and various types of carriers such as gels, hydrogels, sponges, microspheres, liposomes, nanoparticles, polymers, etc., preferably biocompatible [5,10,11,12,13,14,15]. An ideal DDS should transport the drug to the delivery site intact and control its release in dose and over time [5,16]. In contrast to passive DDS, active delivery systems allow for adjusting the released amount of the drug through external physical or chemical stimuli. These include ultrasound, light, electric, and/or magnetic fields [17,18,19,20], mechanical forces, temperature, or pH [10,12,21]. As an example, drug delivery devices (DDDs) remotely activated by a magnetic field have been recently reported [22,23]. These devices are based on the deformation of a responsive polymer induced by a magnetic stimulus, leading to the release of the drug in the surrounding medium. In general, polymers have been recognized as the most promising candidates for developing controlled release devices for clinical applications [6,24]. Thus, the advent of novel responsive polymers will extend the possibilities for developing cost-effective DDDs with extended capabilities.

**Figure 1:**
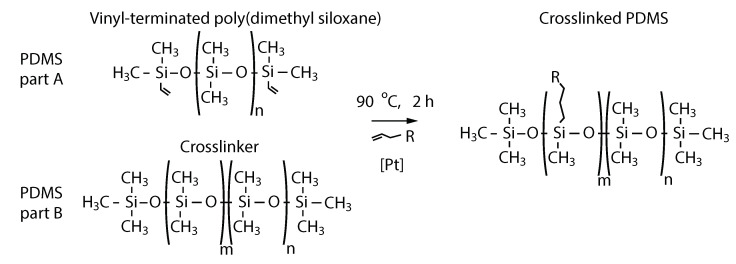
Structure of the polydimethylsiloxane (PDMS): pre-polymer (PDMS part A) and crosslinker (PDMS part B). The crosslinked structure of the polymer (right) is obtained after the alkane reacts with the silicon hydride under the influence of the platinum as a catalyst [35].

Polydimethylsiloxane (PDMS) is a widely used polymer in different applications due to its remarkable thermal, mechanical, and biological properties [25,26,27]; its molecular structure is shown in Figure 1. It can be further converted into a responsive polymer upon combining it with different micro- or nanoparticles [28,29,30,31]. In DDS-related applications, a PDMS capsule with embedded iron particles has been shown to provide drug releasing capabilities upon excitation with a magnetic field [31]. However, the dose released by these magnetic DDS depends on the intensity of the magnetic field and this imposes some restrictions on the stability of the magnetic source. Since light sources offer several means to achieve accurate control of their spatial and temporal features, the use of light to activate DDS renders an attractive alternative to magnetic fields. Albeit limited by its wavelength-dependent penetration in tissue, light-activated DDS have shown promising results [32]. Based on PDMS nanocomposites incorporating gold nanoparticles or carbon nanotubes, micro-pump systems have been demonstrated as devices with drug releasing capabilities [13,33,34]. These devices operate upon deflection of a membrane of the PDMS nanocomposite, achieved via near-infrared (NIR) light absorption by the nanomaterials.

Efforts towards developing light-activated DDDs based on PDMS composites have focused on using expensive nanomaterials (e.g., gold, graphene), and their design has relied on the deflection of a membrane. Nonetheless, more accessible nanomaterials yielding nanocomposites with enhanced light-driven features may provide a building block for DDS with enhanced designs and improved performances. Herein, we report on the development of a drug delivery device based on novel design and a photoresponsive PDMS nanocomposite, showing the feasibility of using this material to develop DDDs. Devices can be fabricated following a simple procedure with drug releasing capabilities relying on a photomechanical effect triggered in the nanocomposite by pulsed NIR irradiation from a laser diode (975 nm). A pair of millimeter-size, concentric PDMS cylinders are used to form the device: while the inner cylinder, fabricated with PDMS and carbon nanoparticles (CNPs), renders an active element that expands thermally upon NIR irradiation, the outer cylinder (blank PDMS) serves as capsule for the device. The gap between both cylinders was filled with a marker solution in order to evaluate the release capabilities of the device. Two PDMS lids were used to seal both sides of the cylindrical capsule, one of them incorporating a micropore serving as the release gate for the marker solution, opening only when the thermal expansion of the active element creates a positive pressure within the capsule. A full characterization of the device, namely, the physicochemical features of the nanocomposite, as well as its thermal, mechanical, and drug delivery responses were evaluated.

## 2. Results and Discussion

### 2.1. Nanocomposites and DDDs

**Figure 2:**
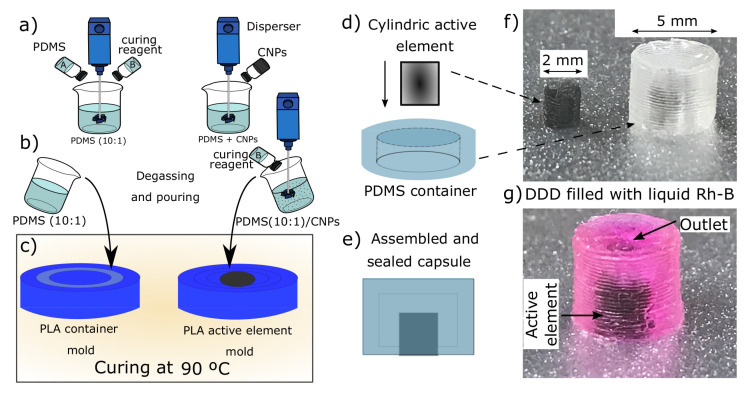
Drug delivery device based on polydimethylsiloxane nanocomposites: (**a**) synthesis of the PDMS/CNPs nanocomposites; (**b**) degassing and pouring of the polymers within the poly(lactic acid) PLA molds; (**c**) curing process to obtain the containers and the active elements; (**d**) assembling of the containers and active elements; (**e**) assembled and sealed capsule ready to be filled with Rh-B; (**f**) image of a real active element and PDMS container; and (**g**) image of a real DDD filled with Rh-B.

The preparation of the nanocomposites and the fabrication of the drug delivery device is illustrated in Figure 2. First, PDMS part A (pre-polymer) and PDMS part B (curing reagent) of the Sylgard 184 PDMS were weighted in a 10:1 ratio, respectively. These were then mixed to obtain the blank PDMS (10:1) blend as shown in Figure 2a. Alternatively, CNPs were first mixed and dispersed in part A; then, the curing reagent was added, and this mixture was blended again to obtain the nanocomposites. This procedure was followed to obtain the nanocomposites with different concentrations of CNPs (0.1% wt., 0.5% wt., 1% wt., and 3% wt.). After mixing, the blank PDMS and the PDMS/CNPs nanocomposites were degassed and then poured into the container and active element molds (see Figure 2b) to be cured in an oven at 90 °C for 2 h, see Figure 2c. Once cured, the containers and the active elements were demolded and assembled concentrically in liquid PDMS (10:1) contained in a Petri dish. These arrangements were subsequently cured in an oven at 90 °C for 2 h until sealing was achieved, see Figure 2d,e. As a final step, the sealed capsules were filled with Rh-B, as is depicted in the images of Figure 2f,g.

### 2.2. Physical and Chemical Features of the Nanocomposites

**Figure 3:**
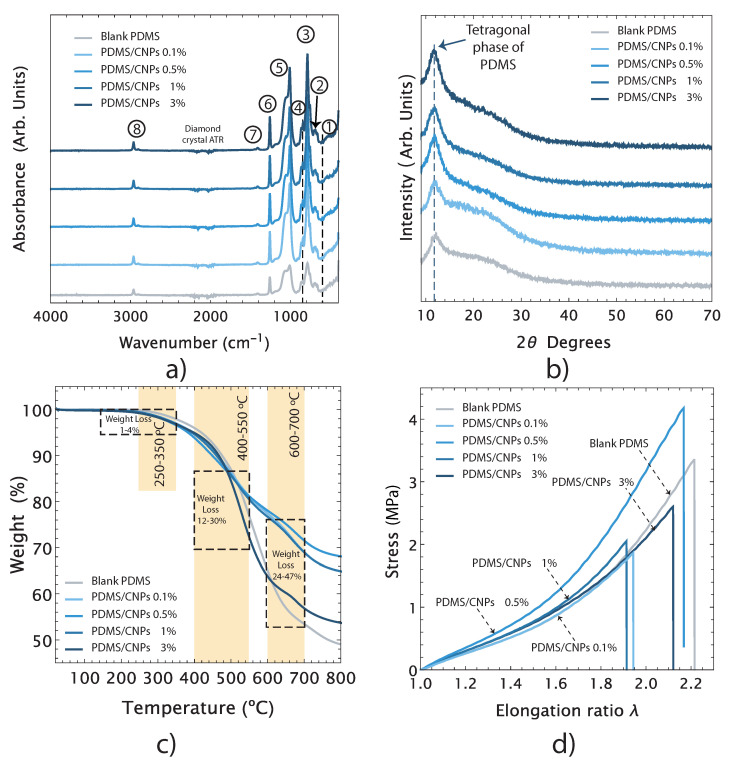
Main physical and chemical features of the nanocomposites: (**a**) chemical features of the nanocomposites determined by FT-IR; (**b**) X-ray diffraction patterns and crystallinity; (**c**) thermal stability by TGA; and (**d**) mechanical behavior under uniaxial tension.

Figure 3a shows the FT-IR spectra of the blank PDMS and its nanocomposites and the bands assignations have been summarized in Table 1. The origins of each of these bands are listed in the table along with the references of previous reports on these features with similar nanocomposites. The registered FT-IR spectra show that the addition of CNPs in the polymer does not significantly affect its chemical structure, suggesting that the interactions between the nanoparticles and the PDMS matrix are only physical. Therefore, the inclusion of CNPs might have an impact on important features of the PDMS/CNPs nanocomposite such as its crystallinity, thermal stability, and/or stiffness.

The X-ray diffraction patterns for blank PDMS and for the PDMS/CNPs nanocomposites (0.1% wt, 0.5% wt, 1% wt and 3% wt) are presented in Figure 3b. All the samples showed a characteristic peak between 2θ = 11.5 − 12${}^{\circ }$, corresponding to the tetragonal crystal lattice of PDMS. The peaks associated with the carbon nanoparticles—peaks around 25${}^{\circ }$ and 43${}^{\circ }$ corresponding to the (002) and (100) planes of graphite, respectively—were not observed in the X-ray diffraction patterns. However, the effect of the CNPs in the crystallinity of the nanocomposites was verified upon evaluating the corresponding areas of the X-ray diffraction patterns for each sample by means of the *cut and weight* approach [36]. The estimated crystallinity values were 18, 20, 30, 35, and 37% for blank PDMS, PDMS/CNPs (0.1% wt, 0.5% wt, 1% wt and 3% wt.), respectively. Clearly, the crystallinity increased along with the content of CNPs (it almost doubled for the last two cases compared to blank PDMS). This enhancement in crystallinity suggests that the CNPs served as nucleation sites where the polymeric chains of PDMS can initiate their folding arrangement, as observed in other polymeric matrices filled with carbon nanoparticles [37]. The relevance of this physical interaction is that an increase in crystallinity yields a decrease in the entropy of the nanocomposite, improving the photomechanical response triggered by absorption of NIR light. The resulting increase in temperature due to optical absorption in turn increases the entropy of the ordered polymeric chains causing a volumetric expansion of the PDMS matrix.

Figure 3c shows the TGA curves for blank PDMS and its nanocomposites showing their thermal stability features. Notice that all samples are stable from room temperature (25 °C) up to temperatures just below 200 °C. The weight loss percentage as a function of temperature occurs mainly in a two-step degradation processes, although we also detected an initial degradation stage with 1–4% of weight loss within a temperature range of 250 and 350 °C, corresponding to the evaporation of loosely bound water and/or residual solvents. As seen in the TGA curves, the first notorious degradation step occurred between 400–550 °C, showing weight losses ranging between 12 and 30% with respect to their original weights. These are due to degradation of the PDMS into cyclic oligomers due to Si-O bond scission [38]. The second notorious degradation step was observed when the PDMS was exposed to higher temperatures, from 600 to 700 °C, yielding a weight loss between 24–47% with respect to their original weights. At these temperatures, the thermal degradation of PDMS begins via depolymerization of the polysiloxane backbone, leading to the formation of cyclosiloxanes [39]. It has also been reported that, for temperatures from 600 °C and above, a radical mechanism occurs throughout the SiCH${}_{3}$ bond scission yielding methane through hydrogen abstraction [40]. The residual weights for the blank PDMS and its nanocomopsites PDMS/CNPs (0.1% wt, 0.5% wt, 1% wt and 3% wt. of CNPs) at 800 °C were 49, 65, 68, 65, and 54%, respectively. In general, the thermal degradation of PDMS can be affected by factors such as solvent and oxidation, end-group functionality, or even impurities. However, the operating temperature range (room temperature to 150 °C) of the proposed drug delivery device falls within the thermal stability range for these nanocomposites and are therefore thermally suitable for our purposes.

Figure 3d shows representative stress vs. elongation ratio curves of blank PDMS and its nanocomposites. All samples exhibited a nonlinear mechanical response under uniaxial tension. Using the experimental data and a second-order Ogden model, the elastic modules were determined as 1.14, 1.26, 1.43, 1.52, and 1.44 MPa for the blank PDMS and for the 0.1% wt, 0.5% wt, 1% wt, and 3% wt PMS/CNPs nanocomposites, respectively. Thus, the mechanical response of the nanocomposites improves upon increasing the concentration of CNPs, agreeing with the increase in crystallinity observed from the X-ray diffraction results. The addition of CNPs hence modified the stiffness of the nanocomposites within a range of 10 to 33% with respect to the elastic modulus of the blank PDMS. Notice, however, that the elastic modulus of the 3% wt. sample suggests that, at this concentration, the CNPs start acting as a stress concentration points—as commonly observed in polymer nanocomposites with nanoparticle inclusions—instead of behaving as a reinforcement agent [41].

### 2.3. Optimization of the Irradiation Optical Powers

**Figure 4:**
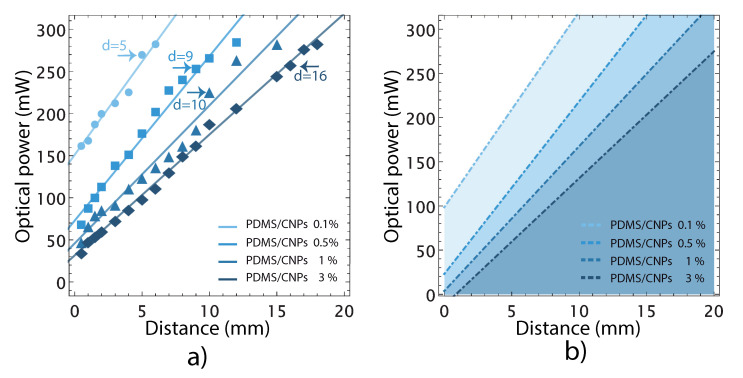
Optical power limits before thermal damage appears by incandescence in the nanocomposites: (**a**) optical power limits associated with their critical working distance for each nanocomposite; and (**b**) representation of safety regions considering a 20% less than the optical power limit for each nanocomposite. These shadowed regions represent safe (optical power and distance) working parameters to avoid incandescence.

The PDMS nanocomposites absorb NIR radiation and under some conditions the increase in temperature can lead to incandescence. Figure 4a presents optical powers for different irradiation distances registered for each sample. The experimental points represent the maximum optical power, for a given distance that the samples can withstand before incandescence. Optical powers beyond these levels damaged the nanocomposites. For each PDMS/CNPs nanocomposite (0.1%, 0.5%, 1% and 3%), we were able to identify an optimum irradiation distance for reaching maximum deformation before incandescence (d = 5, d = 9, d = 10, d = 16 mm, respectively), as marked in Figure 4a. As shown in the plot, larger irradiation distances are allowed for the nanocomposites with higher CNPs concentration to observe the photomechanical effect. Using these results, we defined the working regions for each nanocomposite upon obtaining a linear fit for the experimental points. The straight lines thus define the optical powers that can be safely used for a given irradiation distance avoiding incandescence. Since some experimental points might fall below these *safety lines*, an additional safety factor was considered using the residuals of the fitting. Hence, the safe working regions shown in Figure 4b were established upon considering irradiation powers 20% below than those originally observed for each nanocomposite. The shaded regions in the figure thus indicate the ranges of optical powers and irradiation distances that would avoid thermal damage and incandescence for each PDMS/CNPs nanocomposite.

### 2.4. Thermal Fluorescence Imaging Characterization by LIFT

**Figure 5:**
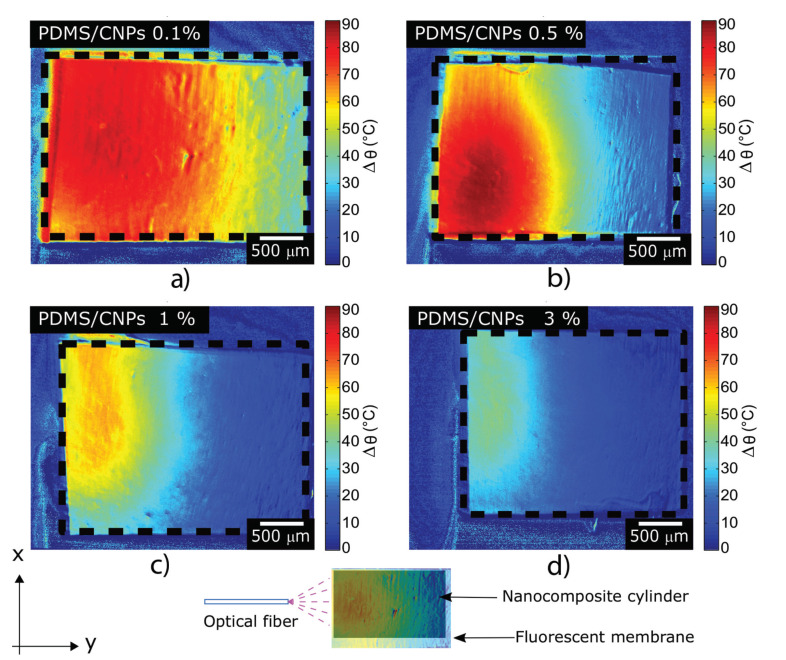
Temperature maps of the nanocomposites undergoing NIR irradiation obtained by LIFT; these figures depict the temperature distributions generated through the NIR absorption of the nanocomposites: (**a**) PDMS/CNPs 0.1% using d = 5 mm; (**b**) PDMS/CNPs 0.5% using d = 9 mm; (**c**) PDMS/CNPs 1% using d = 10 mm; and (**d**) PDMS/CNPs 3% using d = 16 mm. Video S1, in the Supplementary Material, shows the evolution of the heating process.

Figure 5a–d depict the steady-state temperature gradient of the materials after irradiation with the NIR diode laser for 300 s; all the nanocomposites reached a steady temperature profile after 90 s of irradiation. Figure 5a shows that the laser effectively interacted with the PDMS/CNPs 0.1% nanocomposite, penetrating approximately 2 mm and thus increasing the temperature up to 70–80 °C across the whole diameter of the cylinder. In contrast, a shorter penetration depth was observed for the PDMS/CNPs 0.5% cylinder (approximately 1 mm, see Figure 5b) owing to the increase in absorption. Notice, however, that the temperature reached an increase of almost 90 °C within a small localized region (0.5 mm diameter) of the cylinder. Meanwhile, the temperature profile of the PDMS/CNPs 1% shown in Figure 5c, exhibited a temperature increase of approximately 50–60 °C and a light penetration of nearly 1 mm. Finally, the nanocomposite with the highest concentration of CNPs (PDMS/CNPs 3%) showed the shortest laser light penetration (around 0.5 mm) and the lowest temperature increase (30–40 °C), as shown in Figure 5d.

From the LIFT results, it is evident that the concentration of CNPs in the PDMS plays a role in the temperature responses for the nanocomposites. As the CNPs concentration increases, the exponential decay of light due to optical absorption also increases and therefore less radiation reaches deeper layers inside the cylinders. This in turn limits the heating zone within the cylinder as the photothermal effect is produced close to the surface (see Figure 5c,d). The temperature distributions for each sample show that an increase in CNPs concentration yields a non-monotonic variation in temperature along the *y*-direction as displayed in Figure 5. Since the temperature distribution is closely linked to the thermo-mechanical stresses developed in the materials and, therefore, to its deformations, we also registered and analyzed the changes in shape of the active cylindrical elements.

### 2.5. Volumetric Deformation and Stress Caused by NIR Irradiation

**Figure 6:**
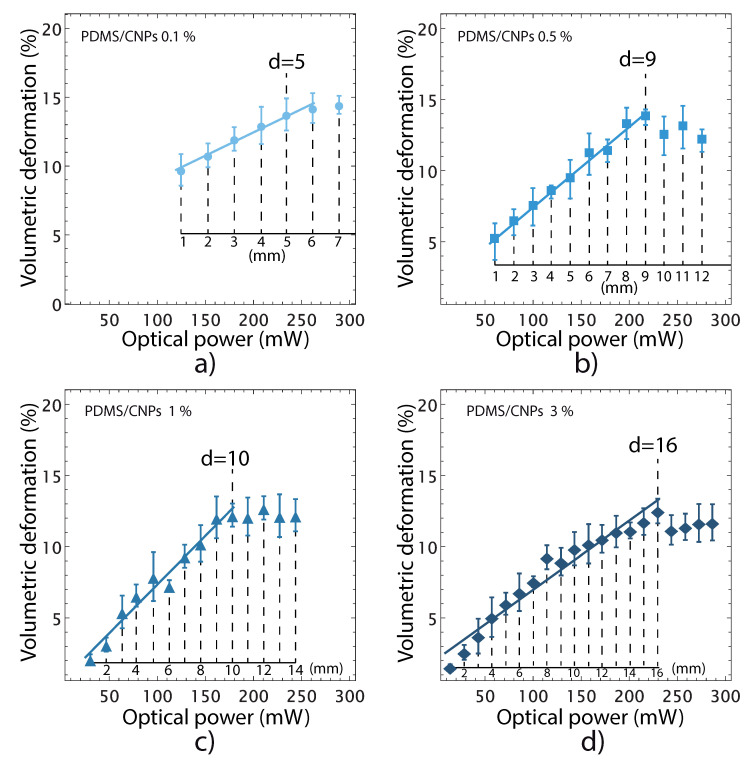
Volumetric deformation of the nanocomposites caused by NIR irradiation. The irradiation distances—used to obtain each point in the curves—are indicated in the inset axes: (**a**) PDMS/CNPs 0.1% showing a maximum deformation with an optimal distance equal to 5 mm; (**b**) PDMS/CNPs 0.5% showing a maximum deformation with an optimal distance equal to 9 mm; (**c**) PDMS/CNPs 1% showing a maximum deformation with an optimal distance equal to 10 mm; and (**d**) PDMS/CNPs 3% showing a maximum deformation with an optimal distance equal to 16 mm.

The changes in shape of the cylinders were evaluated through image processing analysis; the optimal irradiation distances of irradiation mentioned in Section 3.4.3 for each nanocomposite were again verified in these experiments. Beyond these optimal distances, nonlinear behaviors were detected in the volumetric deformation vs. optical power curves for each nanocomposite, shown in Figure 6.

In addition, these results showed that the nanocomposites exhibit volumetric deformations ranging from 12 to 14%; the nanocomposites with the lowest (largest) concentration of CNPs yielded the largest (smallest) volumetric deformations. This result shows that large concentrations of CNPs reduce the penetration depth of the laser light owing to an increase in absorption. Hence, further light interaction with the CNPs is inhibited, thereby reducing the deformation capabilities of the nanocomposites.

**Figure 7:**
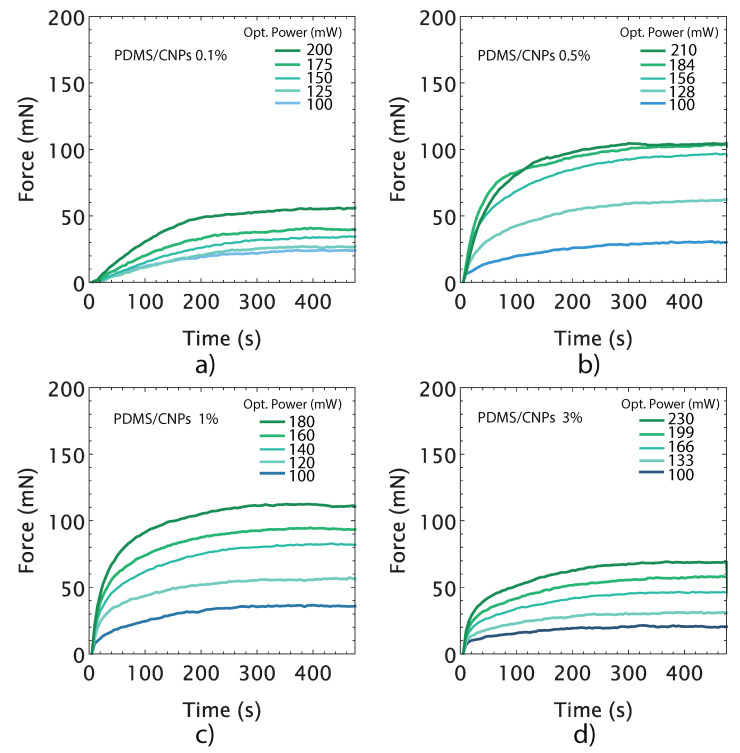
Optically driven forces produced by nanocomposites as a function of time for different optical power outputs: (**a**) PDMS/CNPs 0.1% considering an optimal distance d = 5 mm; (**b**) PDMS/CNPs 0.5% considering an optimal distance d = 9 mm; (**c**) PDMS/CNPs 1% considering an optimal distance d = 10 mm; and (**d**) PDMS/CNPs 3% considering an optimal distance d = 16 mm.

Along with the volumetric deformation, the active elements—i.e., the cylinders of nanocomposites—also provide actuation forces through photomechanical effects. These were evaluated using five different optical powers for each active element considering their corresponding optimal working distances as determined in Figure 6. The force vs. time curves represent the force delivery capacity of the nanocomposites at different optical power (see Figure 7). As the laser power was increased, the optically driven force (ODF) generated by the nanocomposite increased accordingly. The largest ODF values were 104 (±0.4) and 112 (±0.6) mN, corresponding to the PDMS/CNPs 0.5% and PDMS/CNPs 1% nanocomposites, respectively (see Figure 7b,c). In contrast, the PDMS/CNPs 0.1% and PDMS/CNPs 3% nanocomposites yielded ODFs of 55 (±0.8) and 68 (±0.6) mN, respectively (see Figure 7a,d). In all cases, the ODFs were obtained averaging the registered forces for NIR irradiation times from 300 to 500 s.

The force vs. time curves also provide information regarding the time response of the active elements. In particular, the slopes of the linear regions of the curves observed immediately after laser irradiation yield the force actuation velocity (in mN per second) for each nanocomposite. Table 2 summarizes the results from this analysis, showing that this feature increased with the concentration of CNPs and also with the optical power. From these results, and using the optimum irradiation parameters for each case (see Table 2), we identified the fastest nanocomposite: the PDMS/CNPs 3%, offering up to 68 mN at a rate of 1.89 mNs${}^{-1}$. In contrast, the slowest nanocomposite was the PDMS/CNPs 0.1% with 55 mN at a rate of 0.33 mNs${}^{-1}$.

**Figure 8:**
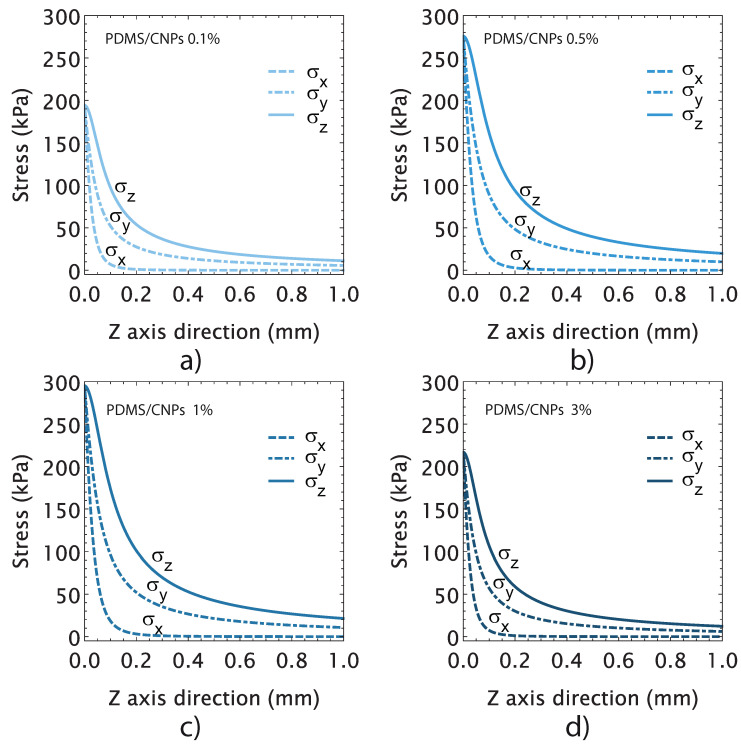
Stress-induced in the cylindrical active elements by NIR irradiation considering the optimal distance and the optimal optical power output for each nanocomposite: (**a**) PDMS/CNPs 0.1% showing the state of the stress through *z*-axis direction considering an optimal distance d = 5 mm and 200 (mW); (**b**) PDMS/CNPs 0.3% showing the state of the stress through the *z*-axis direction considering an optimal distance d = 9 mm and 210 (mW); (**c**) PDMS/CNPs 1% showing the state of the stress through the *z*-axis direction considering an optimal distance d = 10 mm and 180 (mW); and (**d**) PDMS/CNPs 3% showing the state of the stress through the *z*-axis direction considering an optimal distance d = 16 mm and 230 (mW).

Following the concepts that are presented in Section 3.4.3, we evaluated the maximum stresses induced on the active elements in order to determine a conversion factor between the optical and mechanical energies. The plots in Figure 8 show the state of stress attained by the active cylinder for each nanocomposite. Unlike the free deformation experiments shown in Figure 6, for these experiments, the active cylindrical elements were placed between compression plates in order to determine the mechanical stresses induced by NIR irradiation. As shown in Figure 8a–d, the thermal expansion produced normal stress components in the *x*, *y*, and *z*-directions ($\sigma_{x}$, $\sigma_{y}$ and $\sigma_{z}$), as it was expected. The plots further show that the normal stresses decayed exponentially until vanishing, reaching a maximum value at the irradiation zone and decreasing at distances of the order of the radius of the cylinder. The maximum values of stresses were registered in the contact region—between the plates and the cylinder—yielding $\sigma_{z}$ values of 194, 275, 295, and 216 MPa for the PDMS/CNPs 0.1%, PDMS/CNPs 0.5%, PDMS/CNPs 1%, and PDMS/CNPs 3% nanocomposites, respectively.

To evaluate the energy conversion factor $\eta_{M}$, we used the registered normal stresses in the *z*-direction. In contrast to previous reports in which a conversion factor for graphene nanocomposites has been defined [46], we considered the effective irradiated energy into the cylinder end face. The effective optical power irradiating the cylinder was thus defined as $OP_{\mathrm{effective}}=\left(\frac{OP_{\mathrm{total}}}{A_{\mathrm{beam}}}\right)*A_{\mathrm{total}}$, where $OP_{\mathrm{total}}$ is the total optical power from the laser diode, and $A_{\mathrm{beam}}$ and $A_{\mathrm{total}}$ are the beam interaction area and the cylinder area, respectively. The conversion factor can then be calculated as $\eta_{M}=\frac{\sigma_{z}}{OP_{\mathrm{effective}}}$, and, in terms of the optical energy, this may be rewritten as $\eta_{M}=\frac{\sigma_{z}}{E_{\mathrm{light}}}$, where $E_{\mathrm{light}}$ is the light energy in Joules, and it is defined as $E_{\mathrm{light}}=OP_{\mathrm{effective}}*t$, where *t* is the irradiation time in seconds. The calculated conversion factors are presented in Table 3 along with other relevant parameters considered for the selection of a nanocomposite for the drug delivery device. Note that the energy conversion factor increased with the content of CNPs. For drug delivery purposes, a nanocomposite capable of producing large force/stress with a moderate increase in temperature is desirable, since this may provide suitable stability conditions for the drug. Thus, considering the parameters shown in Table 3, the PDMS/CNPs 1% nanocomposite was selected to conduct the Rh-B delivery experiments. This nanocomposite produced the largest optically driven force (112 mN) and a stress of 295 MPa using only 4.6 J of light energy. The PDMS/CNPs 1% can thus be considered as the most suitable nanocomposite to obtain a functional drug delivery device.

### 2.6. Drug Delivery Evaluation of PDMS/CNPs 1%

**Figure 9:**
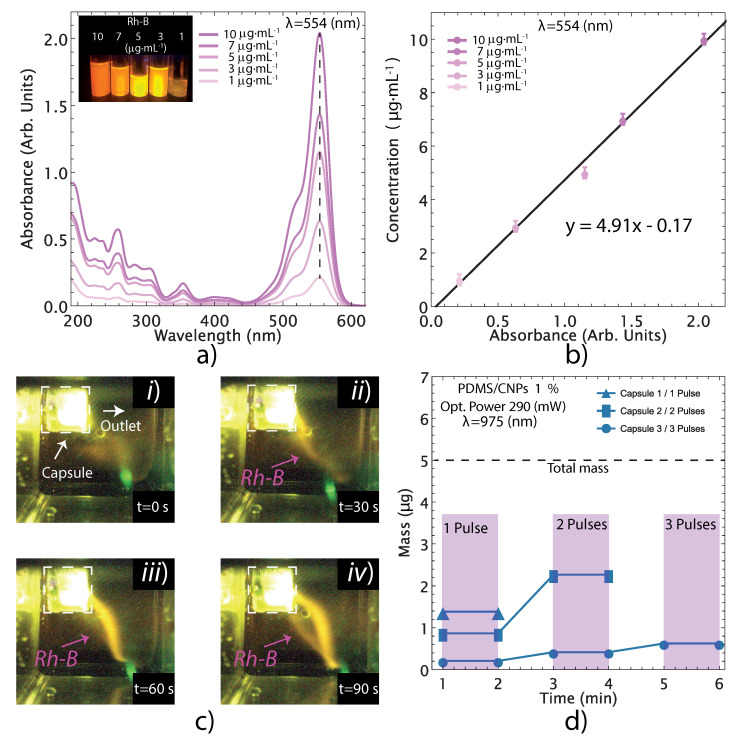
Evaluation of the liquid delivery from the devices using Rh-B as a fluorescent marker for visualization/quantification purposes: (**a**) UV-Vis spectra for Rh-B at different concentrations exhibiting its main peak at λ = 554 nm; (**b**) linear fitting of the concentration vs. absorbance data to obtain the calibration model; (**c**) visualization of Rh-B release under fluorescent imaging; one single long pulse for 90 s; and (**d**) quantification of Rh-B release through UV-Vis measurements. The release of the Rh-B from the device can be visualized in Video S2 of the Supplementary Material.

The drug delivery experiments were conducted only for the PDMS/CNPs 1% nanocomposites because it showed the best responses under NIR irradiation. Aside from providing the largest optically driven force (112 mN) and stress (295 MPa), this nanocomposite also showed a moderate increase in temperature (50–60 °C, in air), exhibiting one of the highest load rates (1.6 mNs${}^{-1}$) compared to the other nanocomposites. Figure 9 shows the performance of the capsule made of PDMS with a cylindrical active element of PDMS/CNPs 1% under pulses of NIR irradiation. Figure 9a shows the UV-Vis spectra of Rh-B dissolved in distilled water at different concentrations (1, 3, 5, 7, and 10 μg·mL${}^{-1}$). These spectra were used to obtain a calibration curve through UV-Vis measurements considering the characteristic peak of Rh-B at 554 nm (see Figure 9a,b). After calibration, the drug delivery experiment was conducted using the optimal parameters of distance and optical power obtained for the selected nanocomposite.

The release of Rh-B contained in the capsule into a UV-Vis cell with distilled water was visualized with the camera and images were captured at different times during the process (see Figure 9c). The content of the cell was subsequently evaluated by UV-Vis spectroscopy to determine the amount of Rh-B released under the NIR irradiation pulses, yielding the plot shown in Figure 9d. The release of Rh-B was evaluated in three different capsules that were previously filled with 15 (μL) of Rh-B solution (with a concentration of 330 μg·mL${}^{-1}$). The first capsule delivered 28% of the total content with a single pulse of NIR irradiation. The second capsule was irradiated with two pulses yielding a 45% Rh-B delivery, and the third capsule underwent three irradiation pulses for a 12% delivery. Notice that the devices do not delivered the whole content at once, but rather expelled volume fractions of the Rh-B according to a specified delivery pattern defined by the laser diode pulse sequence. These results therefore demonstrate that the proposed capsules can deliver fractions of their content for each pulse of NIR laser irradiation. Evidently, the release of the fluid varies for each capsule because of variations of the fabrication process. This process is done by hand, and it is susceptible to misalignment between assembled cylindrical parts and variation of air micro-bubbles reminiscences. The only way to improve reproducibility at the micron scale on different batches is perhaps by using semi-automatic micromanipulators or robotics (as it is done in industrial assembly lines). Nonetheless, our results demonstrate the feasibility of fabricating drug delivery devices with the proposed composites and using the capsules’ geometrical features. As explained in the following section, factors such as the sealing of the capsule, the size of the outlet aperture, and small air bubbles trapped within the capsule can affect the fluid release performance. The capsule design may thus be improved upon in order to obtain increased effectiveness and control in the release of drugs.

#### Numerical Simulation of the Drug Delivery Device

In order to improve the design of the capsules and to understand the mechanisms involved in the drug release, some numerical simulations were conducted. As explained in the supporting information, all the parameters used for the numerical simulations were obtained from the experimental characterization of the nanocomposites. The relevant parameters were mostly reported in the previous sections and others were obtained from well-known standard tables. The only parameter that was fitted after comparing the experimental and numerical volumetric deformations of the PDMS/CNPs active element was the so-called photothermal conversion efficiency, $\eta_{eff}$, used in the absorption model (Lambert–Beer type). After running some preliminary simulations (see the supporting information) we calibrated the governing equations using a value of $\eta_{eff}$ = 0.63 which was then used for the rest of the calculations. We also used a value of $P_{o}$ = 179.1 mW for the optical power, which is the maximum permissible power to avoid strong optical nonlinearities (see the supporting information). The material parameters used for the simulations were those of the capsule fabricated with the 1.0% PDMS/CNPs nanocomposite and used for the Rh-B delivery experiments. As shown in Table 3, this nanocomposite exhibited the best photomechanical features for the drug delivery purposes.

**Figure 10:**
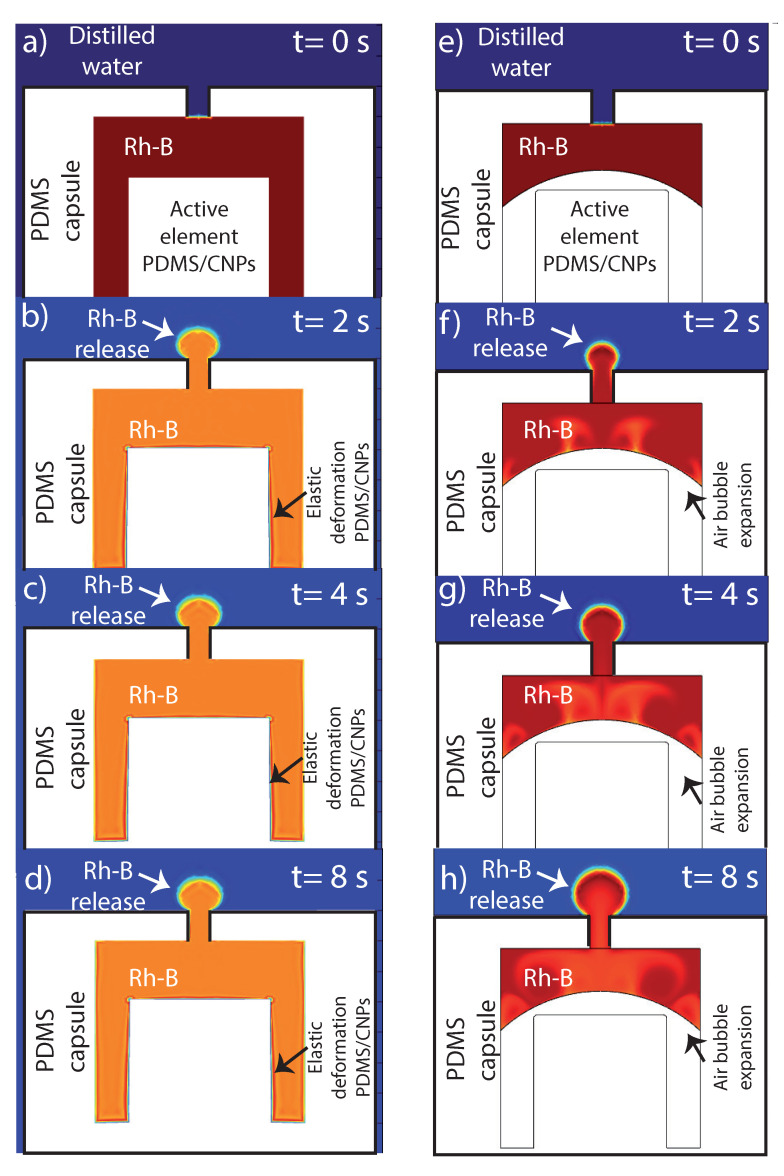
Numerical results explaining the internal phenomena that possibly occurs within the capsule during the drug release: (**a**–**d**) images corresponding to elastic deformation of the nanocomposite at different times; and (**e**–**h**) images simulating the presence of an air bubble that aided to the expulsion of Rh-B under NIR irradiation of the nanocomposite. In order to visualize the evolution of the Rh-B release by numerical simulations, Videos S3 and S4 are available in Supplementary Materials.

The main numerical results are shown in Figure 10a–d. In the first simulation (left column), we emulated the release of marker solution as a function of time (denoted with red color at t = 0) owing to the elastic thermal expansion of the PDMS/CNPs element activated by photothermal conversion. The sequence of images (up to 8 s) clearly show that the elastic deformation of the capsule can promote, by itself, the release of Rh-B initially contained inside the capsule. In addition, an interesting insight provided by the numerical results is that the release of Rh-B by such elastic mechanical stress occurs relatively fast and tends to stabilize or saturate after a certain time; this can be seen in Figure 10a, where the marker travels the orifice in around 1 s and then forms a spherical-like domain that does not grow significantly after that time period.

In the second series of simulations presented in Figure 10e–h (right column), we studied the release of the marker solution, this time by the isobaric expansion of a trapped air bubble in the absence of any solid elastic deformation. As mentioned in the experimental results, air bubbles produced by the assembly of the capsule or during the loading of the marker solution were trapped inside the cavity. A relevant question was therefore if these trapped bubbles can contribute to the release of the capsule’s content. The sequence of time snapshots in Figure 10e–h shows that the isobaric expansion of a trapped air bubble due to the photothermal conversion and further temperature increase of the whole device can also promote the release of the marker solution. Furthermore, in contrast to the solid elastic expansion, the release of the content by bubble expansion can sustain a continuous increase of the released marker solution throughout the whole period of time (8 s). Therefore, bubble expansion does not seem to reach a rapid saturation, thereby sustaining the marker solution release beyond the limits observed for the case of pure elastic deformation. Experimentally, both mechanisms can be present and thus contribute to the short and long-time release process of fluids contained in the proposed device. Furthermore, bubbles may be generated as well during the heating process due to micro-thermal cavitation thus enhancing the marker solution release process [47].

We also tested numerically the possibility that pure marker solution convection, triggered by local density gradients (buoyancy effects), could promote the release of the capsule’s content. These simulations suggest (see supporting information) that pure marker solution convection is not capable of promoting marker solution release by itself and may only help or complement, up to some extent, the two other mechanisms already described above: marker solution release by elastic-thermal deformation and/or marker solution release by isobaric expansion of air bubbles. In any case, these mechanisms provide the required effects to control the release of the marker solution contained in the device.

Evaluation of the device for actually delivering a specific drug should consider the impact of the photothermal, thermal sensitivity, and permeability effects these may have on the drug. The temperature conditions that drugs and drug delivery should be capable of withstanding is a topic of interest for developing DDDs. Temperature effects are in general related to the physicochemical features of the drug itself, and reports have shown that, while some drugs can sustain their features over a wide range of temperatures, some others disintegrate by freezing [48]. Thus, appropriate temperature ranges for operating the DDDs must be defined accordingly. Similarly, the potential effects of any residual near-infrared (NIR) radiation on the drug will depend on the physicochemical features of the medication. It is worthwhile noticing that this wavelength range has been sought as a viable alternative to other wavelengths for drug delivery owing to its deeper transdermal penetration [49,50]. In this sense, the proposed DDDs may offer an advantage over other light-triggered devices. Finally, any possible issues related to gas permeability can be addressed by varying the mixing ratio between oligomers and curing agent of the PDMS. It has been shown that, under some mixing conditions, it is possible to strongly influence the chemical and mechanical properties of the elastomer resulting in a large increase in the permeation of gas molecules across a PMDS membrane [51]. PDMS gas permeability can thus be adjusted as required once a specific drug is selected for delivery.

Finally, we have to comment on the difficulties and challenges of our designed DDDs. Among them, we found that the linear relationship between mechanical deformation and optical power has a limit or plateau; therefore, an increase in optical power does not necessarily translate into an increase in the released volume. However, bubble expansion can alleviate the limitation of the mechanical expansion. As mentioned earlier, reproducibility of the assembly process of the capsule is a challenge that could be overcome by establishing an automated process to achieve this task. This may provide a better approach for obtaining capsules capable to deliver a consistent amount of the drug for each pulse of the NIR diode laser. It is also interesting to compare the advantages that the proposed DDDs may offer when compared to other devices. In general, there is always a trade-off when selecting a specific approach for activating the device. As an example, light intensity attenuation in real biological tissues can be greater than magnetic field attenuation. Hence, depending on the specific application, other DDS such as magnetic responsive devices could reach deeper tissue layers than the optically driven devices proposed here. However, magnetic devices might be orientation dependent and this may impose some restrictions for some types of applications. An interesting feature of our DDDs is that they can be classified among those working with light guided technologies, e.g., fiber optics and other waveguide devices. These are currently used in different areas of medicine such as optogenetics and photodynamic or interstitial therapies [52,53]. Thus, the combination of these light guided technologies with our DDDs could provide synergistic benefits that could enable localized medical treatments.

## 3. Materials and Methods

### 3.1. Materials and Nanocomposites Fabrication

The nanocomposites were elaborated using Dow Corning Sylgard 184 PDMS in a 10:1 ratio and carbon nanoparticles (particle size < 100 nm, Sigma Aldrich, St. Louis, MO, USA, CAS:633100). The blends were prepared as reported in our previous work [30]. Four different concentrations of CNPs 0.1%, 0.5%, 1%, and 3%—in weight—were used to obtain the PDMS nanocomposite blends. The dispersion of CNPs was done at room temperature through mechanical stirring at 2800 RPM for 5 min with a subsequent increase in speed to 3600 RPM for 8 additional minutes as previously reported [30,41]; subsequently, with the four different blends, the components of the final capsule’s body (membranes and cylinders) were fabricated. The PDMS/CNPs blends were also characterized physicochemically as explained in Section 3.3.

### 3.2. Membranes, Cylinders, and Capsule Fabrication

**Figure 11:**
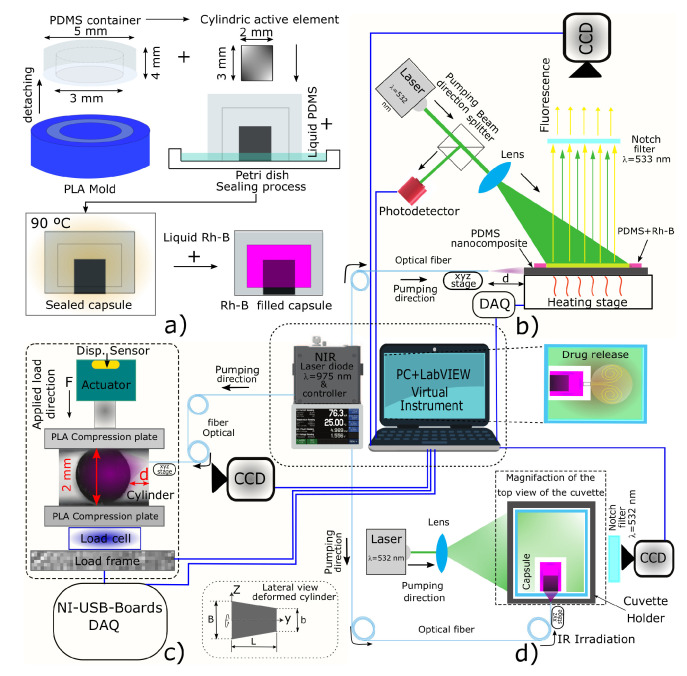
General overview of the different experimental setups to evaluate the photomechanical response of the nanocomposites: (**a**) device fabrication process and mold details; (**b**) laser-induced fluorescence thermometry (LIFT) measurements of the nanocomposites undergoing NIR irradiation; (**c**) NIR laser stress/strain-induced within the nanocomposites; and (**d**) drug release experiment triggered by a photomechanical effect.

To fabricate the PDMS membranes and cylinders, the curing reagent was added in a 10:1 weight ratio, and each blend was subsequently mechanically mixed for 5 min. The cylindrical active elements were obtained upon pouring the PDMS/CNPs nanocomposites into PLA cylindrical 3D printed molds (2 mm internal diameter and 3 mm depth), while the membranes were obtained using Petri dishes to allocate blank PDMS. Both elements were cured at 90 °C for 2 h and after cooling, they were demolded and assembled concentrically in a Petri dish containing liquid PDMS (see Figure 11a). This arrangement was then finally cured at 90 °C for 2 h to obtain a sealed capsule ready to be filled with rhodamine B (Rh-B) for the drug delivery experiments.

### 3.3. Physicochemical Characterization

The physicochemical characterization was performed by means of Fourier transform infrared (FT-IR) spectroscopy, X-ray diffraction, thermogravimetric analysis, and uniaxial tensile tests.

FT-IR was used to identify the polymeric chemical groups and their possible interaction between the blends components and carbon nanoparticles. The FT-IR measurements were conducted on dry membranes with thicknesses close to 500 μm. Attenuated total reflectance (ATR) was used considering the following parameters: wavenumber range from 4000–400 cm${}^{-1}$, 32 scans, and a resolution of 2 cm${}^{-1}$ (Nicolet 6700, Thermo Fisher Scientific, Waltham, MA, USA).

The crystallinities of the nanocomposites were obtained by X-ray diffraction with an X-ray diffractometer (Bruker D8 Advance, Billerica, MA, USA) operating at 45 kV with Ni-filtered Cu K$\alpha_{1}$ radiation (λ = 1.5406 Å). The diffraction patterns were recorded over the 2θ range of 10 to 70° with a scan rate of 0.4 °min${}^{-1}$. The crystallinity percentage was calculated using a *cut and weight* approach as reported in the literature [37].

The thermal stability and decomposition rate of the nanocomposites were evaluated by thermogravimetric analysis (TGA Q5000 IR from TA Instruments, New Castle, DE, USA.) using a nitrogen mass flow rate of 25 mL min${}^{-1}$ and a temperature rate of 10 °C min${}^{-1}$. The TGA data were analyzed using the TA Instruments Universal Analysis 2000 software (TA Instruments, New Castle, DE, USA).

The tensile test was carried out in a custom-designed mechanical tester; the membranes were cut with a special jig and following the ASTM D1708 standard. The tensile test were conducted at rate of 0.16 mm s${}^{-1}$. Time, force, and displacement data were registered by a computer for further analysis. The analysis involves the obtention of the stress (σ) vs. elongation (λ) curves for each specimen. Then, the Ogden model for simple tension [54] was used to analyze the experimental data following the mathematical treatment previously reported in [41].

### 3.4. Performance of the Drug Delivery Device

The experiments to evaluate the performance of the drug delivery device included laser-induced fluorescence thermometry (LIFT) measurements, evaluation of the volumetric deformation caused by laser infrared irradiation, and the drug delivery capacity of the device triggered by a photomechanical effect.

#### 3.4.1. Determination of Critical Parameters before Thermal Damage of the Nanocomposites

The laser irradiation distance (d) to avoid thermal damage (i.e., incandescence) of the nanocomposites was first determined for each nanocomposite. We used a fiber-coupled NIR laser diode (λ = 975 nm, 5–290 mW), and the distance (d) between the fiber and the nanocomposite was varied using a linear translation stage. For each distance, the output power of the laser diode was varied and the thermal damage—owing to an increase in temperature within the nanocomposite—was determined visually using a CCD camera. The combination of these parameters before thermal damage were thus found for each nanocomposite. These results were then represented in a power output vs. distance plot showing the safety regions of irradiation (indicated as shaded areas in the plot) to avoid incandescence of the nanocomposites.

#### 3.4.2. Laser-Induced Fluorescence Thermometry (LIFT)

A LIFT technique was used to obtain the temperature distribution within the cylinder. The temperature of the samples was adjusted with a PID controller and a CCD camera with a notch filter were used for acquiring the temperature dependent fluorescence intensity. Further details of the LIFT system can be found elsewhere [55]. A data acquisition (DAQ) system is connected to a PC where a virtual instrument (VI) programmed in LabVIEW was used to simultaneously control and acquire data and images of the experiment (see Figure 11b). For this characterization, the nanocomposite cylinder was sliced at half and covered with a membrane based on PDMS and Rh-B. This fluorescent and temperature-sensitive membrane was used to observe the temperature gradient on the flat surface of the cylinder. The experimental setup used for these purposes is shown in Figure 11b, and the methodology, calibration, and image processing have been described by Gonzalez-Martinez et al. [55]. This process yields images of the temperature maps corresponding to the surface of the cylinder that is irradiated with an NIR laser diode (λ = 975 nm, 5–290 mW).

#### 3.4.3. Evaluation of the Volumetric Deformation and the Stress Caused by Laser Infrared Irradiation

Figure 11c depicts the experimental setup used to evaluate the volumetric deformation and the stresses caused in the nanocomposite through NIR laser irradiation. To estimate the volumetric deformation due to the photothermal effect, the active cylindrical element, which is the main part of the capsule, was placed on the lower compression plate removing the upper plate, see Figure 11c. The optical fiber—coupled to the NIR laser diode—was moved about the cylinder using a translation stage until reaching the desired distance (d). A CCD camera was placed on one lateral side of the cylinder to visualize and register the changes in the length of the cylinder under NIR irradiation. These changes were evaluated through image processing analysis using ImageJ software –from National Institutes of Health and the Laboratory for Optical and Computational Instrumentation (LOCI, University of Wisconsin, USA). In this series of experiments and after 150 s of NIR irradiation, we noticed that the shape of the cylinders resembled a truncated cone owing to the light penetration and absorption. Hence, given the detected heterogeneous strains, the critical segments of the cylinder experiencing changes in length were determined. These segments—depicted in the right lower corner of Figure 11c—were the length of the cylinder/truncated cone (L), the diameter of the cylinder (b) that also corresponds to the minor diameter of the truncated cone, and the major diameter (B). Their corresponding increase in lengths ($\delta_{L}$, $\delta_{b}$, and $\delta_{B}$) were estimated by digital image analysis. Now, considering the strain engineering equation and the integral of volume for a truncated cone, this yields an expression (Equation (1)) to determine the percentage of volumetric deformation of the active element:

(1) $$V\left(\%\right)=\left(\delta_{L}+\frac{1}{3}\left(\delta_{b}^{2}+\left(3+\delta_{B}\right)*\left(\delta_{b}+\delta_{B}\right)\right)*\left(1+\delta_{L}\right)\right)*100.$$

To obtain the stress induced by NIR irradiation and the optically-driven force (ODF) produced by the cylinder, additional elements were added to the experimental setup. Specifically, we added a load frame with a linear actuator as well as force and displacement sensors (see Figure 11c). For these measurements, the cylinder was confined between the compression plates of the mechanical tester using a preload of 10 mN; subsequently, the confined cylinder was irradiated with the laser diode during 500 s to produce thermal expansion. Thus, the expansion produced forces were registered by the force sensor (load cell omega LCFL, 10 N); these were monitored and registered by the PC through the DAQ system and the virtual instrument. Measurements were performed for the four different blends of nanocomposites using five different optical powers with the fiber located at the appropriate distance (d) yielding the maximum volumetric deformation.

With the registered force, the cross-section area of the cylinder, and considering the Hertz contact theory for cylinders [56], the state of stress in the cylinder along the compression axis *z* was determined using Equations (2)–(5):

(2) $$\begin{array}{c} \sigma_{x}=-2\nu P_{\max}\left(\sqrt{1+\frac{z^{2}}{a^{2}}}-\left|\frac{z}{a}\right|\right), \end{array}$$

(3) $$\begin{array}{c} \sigma_{y}=-P_{\max}=\frac{1+2\frac{z^{2}}{a^{2}}}{\sqrt{1+\frac{z^{2}}{a^{2}}}}-2\left|\frac{z}{a}\right|, \end{array}$$

(4) $$\begin{array}{c} \sigma_{z}=\frac{-P_{\max}}{\sqrt{1+\frac{z^{2}}{a^{2}}}}, \end{array}$$

(5) $$\begin{array}{c} a=\sqrt{\frac{\frac{2F(1-\nu_{1}^{2})}{E_{1}}+\frac{\left(1-\nu_{2}^{2}\right)}{E_{2}}}{\frac{2}{d/2}}}, \end{array}$$

In these expressions, $P_{\max}$ is obtained as $P_{\max}=\frac{2F}{\pi aL}$; *F* corresponds to the force values that the cylinder can generate under NIR irradiation; *z* is a distance with values ranging between 0 and the radius (d/2) of the active element, *L* is the length of the cylinder and *a* is determined with Equation (5), in accordance with the Hertz theory. In Equation (5), *E* and ν correspond to the elastic modulus and Poisson’s ratio; meanwhile, subindices 1 and 2 are related to the type of material. In this case $E_{1}$ = 3.5 GPa, $\nu_{1}$ = 0.35 [57,58] for the compression plates made of PLA (see Figure 11), and it has been reported that the elastic modulus of PDMS ($E_{2}$) shows values ranged between 1 to 2 MPa and $\nu_{2}$ = 0.5 for PMDS and its nanocomposites [41,59]. In our case, the value of $E_{2}$ was experimentally determined by the uniaxial tensile test. This analysis yields the curves for the state of stress vs. position along the *z*-axis direction.

#### 3.4.4. Drug Delivery Capacity of the Device Triggered by Photomechanical Effects

Once the different nanocomposite blends were characterized, and once the photomechanical effect was observed and evaluated in the nanocomposites, drug delivery experiments with the assembled capsule-shaped device were carried out. For these experiments, only the nanocomposite that offered the most suitable photomechanical features were used for the active element of the device. The capsules were filled with 15 μL of Rh-B solution (with a concentration of 330 μg·mL${}^{-1}$) by means of an insulin syringe. Then, the loaded capsule was immersed into distilled water contained in a UV-Vis cuvette placed into a four port cuvette holder where the light sources (NIR and green lasers), a notch filter, and a CCD camera were mounted as depicted in Figure 11d. The green laser (λ = 532 nm) was turned on to excite the fluorescence of the Rh-B solution and subsequently the NIR laser was activated during 90 s to irradiate the active element of the device. This caused an increase in temperature yielding thermal expansion of the active element together with an increase of the internal pressure of the capsule. These effects allow for the release of the Rh-B into the distilled water within the cuvette, and the discharge was registered by fluorescence imaging with the CCD camera. After the Rh-B was released from the capsule, the cuvette was removed from the holder to be analyzed by UV-Vis spectrophotometry. Notice that a calibration of concentration curves was previously done to quantify the Rh-B release. Since Rh-B delivery is done by cycles turning on/off the NIR laser, this UV-Vis quantification was performed between each cycle.

#### 3.4.5. Numerical Simulation of the Drug Delivery Device

In order to have a deeper understanding of the mechanisms involved in the release of the marker solution (Rh-B) observed in the drug delivery experiments, we also conducted 2D axisymmetric numerical simulations using COMSOL Multiphysics. In these simulations, we tried to include and couple most of the physics involved in the functionality of the device, starting with the heat generation in the active PDMS/CNPs element due to photothermal conversion. We further considered the mechanical expansion triggered by the rise in temperature of the material, and the release of the marker solution due to the reduction of the inner cavity volume. We simulated as well other effects that may be involved in the release mechanisms (motivated by experimental observations, see further details in the results section) such as possible convective flows generated by local density gradients (buoyancy effects), or the release of the marker solution boosted by the expansion of air bubbles trapped inside the device. A full description of the governing equations, constitutive laws, and boundary conditions implemented in COMSOL can be found in the Supplementary Information Text S5.

## 4. Conclusions

We have demonstrated that the combination of photothermal and photomechanical effects is useful for developing drug delivery devices (DDDs) triggered by NIR pulses. The PDMS/CNPs nanocomposites used in our experiments were capable to convert NIR light into mechanical energy producing displacements and forces within a cylinder fabricated with these materials and contained within a PDMS capsule. While the measured displacements were within dozens of microns, the forces produced by these active elements were on the order of hundreds of millinewtons (mN). As demonstrated, these features effectively allowed for expelling an Rh-B solution out from the capsule. Among the different tested nanocomposites, the PDMS/CNPs 1% wt. exhibited the most promising photomechanical features for DDDs; these include a temperature range of 50–60 °C (in air), an elastic modulus of 1.52 MPa, a volumetric deformation of 12.2%, an optical driven force of 112 mN, an optically stress-induced of 295 MPa, and a load rate of 1.6 mNs${}^{-1}$. From our experimental observations using Rh-B as a marker solution, we can conclude that marker solution delivery from the capsule is dependent on the cylinder deformation, the optically driven force, and the stress generated in the active cylinder, as well as on the increase in temperature under NIR irradiation. Results from numerical simulations suggest that the mechanisms involved in the Rh-B delivery from the device are the elastic thermal expansion of the PDMS/CNPs cylinder activated by photothermal conversion, and an isobaric expansion of air bubbles trapped within the capsule. Evidently, both mechanisms can operate in a synergistic way favoring the release of substances from the capsule. Although further investigation is required to optimize the operation of the proposed device, the reported features are clearly attractive for developing light-activated DDDs for long-term medical treatments.

## Figures and Tables

**Table 1:** Characteristic vibrational bands of PDMS and its nanocomposites.

Peak in Figure 3a	IR Region (cm${}^{-1}$)	Description	Similar Results
1	600	Stretching of Si–C	[42]
2	699	Stretching of Si–O–Si	[42]
3	785–815	CH${}_{3}$ Rocking and Si–C stretching in Si–CH${}_{3}$	[43]
4	875–920	Si–O stretching in Si–OH	[43]
5	1055–1090	Asymmetric stretching of Si–O–Si	[43]
6	1256–1269	Symmetric deformation CH${}_{3}$ in Si–CH${}_{3}$	[42,43]
7	1410	Si–CH=CH${}_{2}$	[42,44]
8	2900–2960	Asymmetric stretching CH${}_{3}$ in Si–CH${}_{3}$	[42,45]

**Table 2:** Optically driven forces (ODFs) and velocities of actuation of the nanocomposites under NIR irradiation located at first times (linear trend regions).

PDMS/CNPs 0.1%, d = 5 mm			PDMS/CNPs 0.5%, d = 9 mm		
**Optical Power**	**Slope**	**ODF**	**Optical Power**	**Slope**	**ODF**
**(mW)**	**(mNs${}^{-1}$)**	**(mN)**	**(mW)**	**(mNs${}^{-1}$)**	**(mN)**
100	0.13	24 ± 0.6	100	0.27	30 ± 0.6
125	0.13	26 ± 0.4	128	0.67	61 ± 0.8
150	0.16	33 ± 0.8	156	1.10	95 ± 1.2
175	0.22	39 ± 0.8	184	1.30	102 ± 0.9
200	0.33	55 ± 0.8	210	1.30	104 ± 0.4
**PDMS/CNPs 1%, d = 10 mm**			**PDMS/CNPs 3%, d = 16 mm**		
**Optical Power**	**Slope**	**ODF**	**Optical Power**	**Slope**	**ODF**
**(mW)**	**(mNs${}^{-1}$)**	**(mN)**	**(mW)**	**(mNs${}^{-1}$)**	**(mN)**
100	0.33	36 ± 0.3	100	0.65	21 ± 0.4
120	0.76	56 ± 0.5	133	0.88	31 ± 0.5
140	1.01	82 ± 0.7	166	1.22	46 ± 0.7
160	1.27	94 ± 0.5	199	1.55	57 ± 0.8
180	1.60	112 ± 0.6	230	1.89	68 ± 0.6

**Table 3:** Main photomechanical features to be considered for devices with PDMS/CNPs nanocomposites. T = Temperature reached after irradiation, E = Elastic modulus, VD = Volumetric deformation, ODF = Optically driven force, $\sigma_{z}$ = Normal stress in z direction, LR = Load rate, LE = Light energy, and CF = Conversion factor $\eta_{M}$.

Content CNPs	T	E	VD	ODF	$\sigma_{z}$	LR	LE $E_{\mathrm{light}}$	CF $\eta_{M}$
(%)	(${}^{\circ }$C)	(MPa)	(%)	(mN)	(MPa)	(mNs${}^{-1}$)	(J)	(kPaJ${}^{-1}$)
0.1%	70–80	1.26	13.74	55	194	0.33	20	9.7
0.5%	80–90	1.43	13.75	104	275	1.3	6.6	41.9
1%	50–60	1.52	12.2	112	295	1.6	4.6	64.7
3%	30–40	1.44	12.5	68	216	1.89	0.9	237.4

## Data Availability

Data are contained within the article.

## Supplementary Materials

The following are available online, Video S1: Temperature maps of the PDMS/CNPs nanocomposites by LIFT, Video S2: Rh-B release by PDMS/CNPs 1% under NIR Irradiation (290 mW), Video S3: Simulation of Rh-B release by elastic deformation, Video S4: Simulation of Rh-B release by a bubble expansion, Supplementary Text S5: Numerical simulations in COMSOL Multiphysics.

## Abbreviations

The following abbreviations are used in this manuscript:

DDDs	Drug Delivery Devices
DDS	Drug Delivery Systems
CNPs	Carbon Nanoparticles
PDMS	Polydimethylsiloxane
NIR	Near Infrared
ODF	Optically Driven Force
