# Supplementary material for: Photomechanical Polymer Nanocomposites for Drug Delivery Devices
Source: Molecules. 2021 Sep 4;26(17):5376. doi: 10.3390/molecules26175376 (PMC8433747; doi:10.3390/molecules26175376)
Supplement: Supplementary file 1 [file molecules-26-05376-s001.zip › SuppMat/SupplementaryText_S5.pdf]

Photomechanical polymer nanocomposites for drug delivery devices

J.D. López-Lugo, R. Pimentel-Domínguez, J. A. Benítez-Martínez, J. R. Vélez-Cordero, J. Hernández-Cordero, F.M. Sánchez-Arévalo

Instituto de Investigaciones en Materiales, Universidad Nacional Autónoma de México

Cátedra CONACyT-Instituto de Física, Universidad Autónoma de San Luis Potosí

August 10, 2021

Supporting Information

Numerical Simulations in COMSOL Multiphysics.

The set of experiments described in the main text were complemented by numerical simulations in order to elucidate, in greater detail, the mechanisms involved in the release capabilities of the proposed device. A full numerical simulation intended to solve all the relevant variables at once would certainly require extensive computational capabilities. Furthermore, some specific settings for the nonlinear solvers required for solving the mathematical problem may not even guarantee numerical convergence. To overcome these limitations, we carried out four independent numerical simulations, each one exploring the effects that a certain set of variables would have in the formulation of the full problem. All the simulations were performed using a 2D axisymmetric domain with physical coordinates $\mathbf{x} = (r, z)$. The main variables to be solved are the temperature T attained in the photothermal composite, the velocity field $\mathbf{u}$ in the liquid, the displacement vector field $\mathbf{y}$ produced by thermal expansion in the elastic PDMS , and the concentration c of a marker solution that will serve only as visual aid to detect the release of the content by the capsule.

We begin by considering the heat transfer equation

$$\rho C_p \left(\frac{\partial T}{\partial t} + \mathbf{u} \cdot \nabla T \right) + \nabla \cdot (-\kappa \nabla T) = Q_{gen} \ , \tag{1}$$

which includes a heat generation term, Q_{gen}, accounting for the photothermal conversion arising from laser absorption by the polymer nanocomposite. Here ρ, C_p and κ are the density, specific heat capacity and thermal conductivity of the material. Assuming a simple model for light absorption (Beer-Lambert law) and a gaussian beam profile, Q_{gen} can be written as:

$$Q_{gen} = \eta_{eff} \kappa_{ext} I_o \text{exp}(-\kappa_{ext} z - r^2/A) \ , \tag{2}$$

where

$$A = w^2/2, \quad I_o = P_o/\pi A \tag{3}$$

and $w = w_o + z\tan\theta$ is the radius of the divergent beam coming out from the optical fiber used for light delivery. The extinction coefficient of the absorbing material is κ_{ext}, and P_o is the optical power; the angle θ is related to the numerical aperture of the optical fiber via $\sin\theta = NA/n_i$, n_i being the index of refraction of the medium; finally, w_o corresponds to the core radius of the optical fiber.

Excluding the value of the so-called photothermal conversion efficiency, η_{eff}, all the parameters included in eqs. 1, 2 and 3 are known, either from the experimental characterization of the composites or from standard tables. In order to estimate η_{eff}, we calibrated the simulations upon comparing the numerical volumetric deformation produced by the thermal expansion of the PDMS/CNPs element with the corresponding experimental values measured and discussed in section 2.4 To do this, the heat equation has to be complemented with a force balance for the cylindric PDMS/CNPs active element that experiences the thermal elastic deformation. Solving for the steady state and in the absence of external volumetric forces, the force balance can be written as (after using the divergence theorem):

$$\nabla \cdot \sigma_{ij} = 0 \ , \tag{4}$$

where σ_{ij} is the Cauchy stress tensor, and the derivatives are computed according to a fixed spatial coordinate frame. According to structural mechanics theory, σ_{ij} can be used only when the initial configuration of the material does not changes significantly or for small rotations [1]. An improved characterization of the mechanical stresses in the material accounting for configurational body changes is provided by the so-called Lagrangian description, based on the initial or referential configuration of the material rather than on the spatial coordinate frame. Eq. 4 can thus be rewritten as

$$\nabla \cdot (F_{ij} S_{kl}) = 0 \ , \tag{5}$$

where $F_{ij} = \mathbf{I} + \nabla \mathbf{y}$ is the deformation gradient tensor and S_{ij} is the second Piola-Kirchhoff stress tensor which for linear, isotropic elastic materials takes the form [1]

$$\mathbf{S} = \mathcal{C}(E, \nu) : \mathbf{E}_{elast} \ , \tag{6}$$

where $\mathbf{E}_{elast}$ is the effective elastic strain given by

$$\mathbf{E}_{elast} = \mathbf{E} - \mathbf{E}_{inelastic} = \mathbf{E} - \alpha(T - T_o) \ , \tag{7}$$

and $\mathbf{E}$ is the total strain given by the Green-Lagrange strain tensor

$$\mathbf{E} = \frac{1}{2}\left[(\nabla \mathbf{y})^T + \nabla \mathbf{y} + (\nabla \mathbf{y})^T (\nabla \mathbf{y})\right] \ . \tag{8}$$

Equation 7 is coupled to the heat equation through the last term, accounting for a thermal isotropic expansion of the PDMS with thermal expansion coefficient α. The elasticity matrix $\mathcal{C}(E, \nu)$, on the other hand, depends on the Young Modulus and Poisson's ratio and can be found elsewhere [2]; it is important to mention that the linear elastic model considered

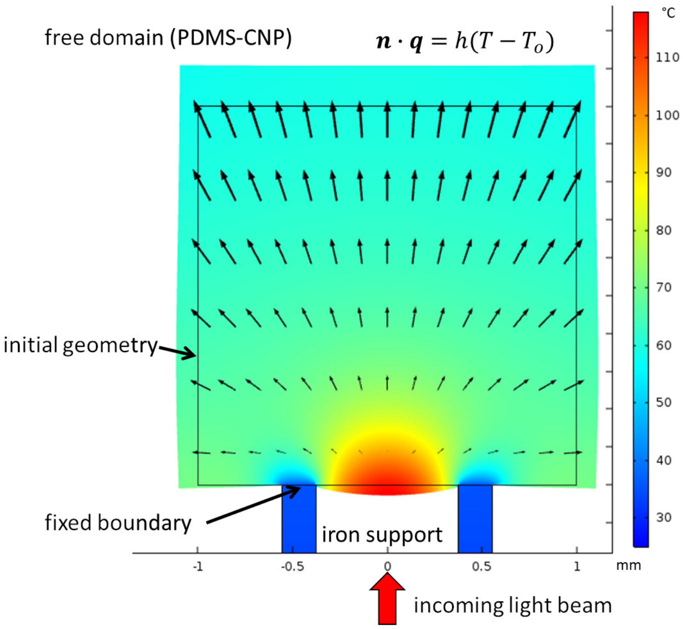

Figure 1: Numerical temperature and steady state displacement fields obtained for the 1.0% PDMS/CNPs composite irradiated with a laser power $P_o = 179.1mW$ and for a selected value of $\eta_{eff} = 0.63$. The initial geometrical configuration is depicted as well as some relevant boundary conditions. The displacement field (arrows) and final configuration is augmented ×3 for visual aid

in these simulations works well for PDMS under strains below 50% (see [3]), which are well below the maximum experimental values reported in this work. Finally, notice that eqs. 5 to 8 reduce to eq. 4 for small displacement gradients of order one in $\nabla \mathbf{y}$.

In the first simulation we solved eqs. 1 and 5 in steady state using the geometry depicted in figure 1 and which represents, geometrically, the 2 × 2 mm PDMS/CNPs active cylinder described in the materials and methods section (notice that for solids, $\mathbf{u} = 0$ in eq. 1). In order to have a well posed problem we allocated fixed geometrical boundaries, as depicted in figure 1, while the rest of the body was left to deform freely. The boundary condition for the heat equation was chosen as a convective dissipative flow of the form $\mathbf{n} \cdot \mathbf{q} = h(T - T_o) = 20[W/m^2K](T - 25°C)$. Figure 1 shows the temperature and steady displacement field of the PDMS/CNPs composite obtained for an optical power of 179.1mW, and using the physical properties of the 1.0% mixture (all the physical parameters used in the simulations are listed in Table 1). Additionally, Figure 2 shows the plot of the experimental and numerical volumetric deformations as a function of the optical power. A photothermal conversion efficiency of $\eta_{eff} = 0.63$ was shown to fit the experimental values quite well up to values of $\sim 200mW$, after which nonlinearities become evident. For the rest of the simulations we therefore used a 179.1mW as a reference for the optical power (the maximum power previous to the appearance of the nonlinear behavior) together with the value of $\eta_{eff} = 0.63$ found in these first simulations.

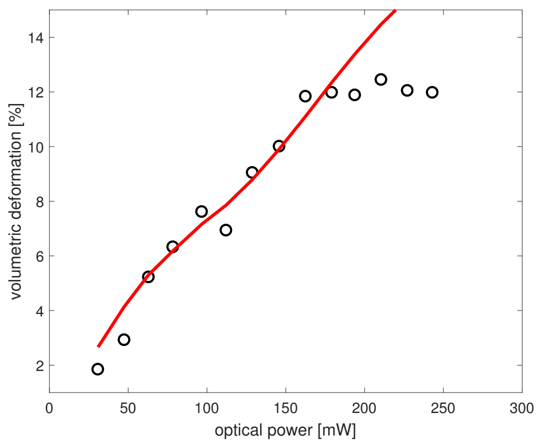

Figure 2: Comparison oh the experimental and numerical volumetric deformations as a function of the optical power; symbols are the experimental data and the line depicts the numerical results. All the simulations were performed using the parameters for the 1.0% CNPs composite.

Fluid-structure interaction model.

A second simulation was carried out for testing the release of liquid from the capsule through thermal expansion of the PDMS/CNPs element. As described in the main text, the expansion of this active cylinder leads to a reduction of the cavity volume thus the marker solution is released. To do this we replicated numerically the experimental setup shown in figure 1d of the main text. Figure 3 shows the 2D axisymmetric version of the capsule together with some of the boundary conditions used in this simulation.

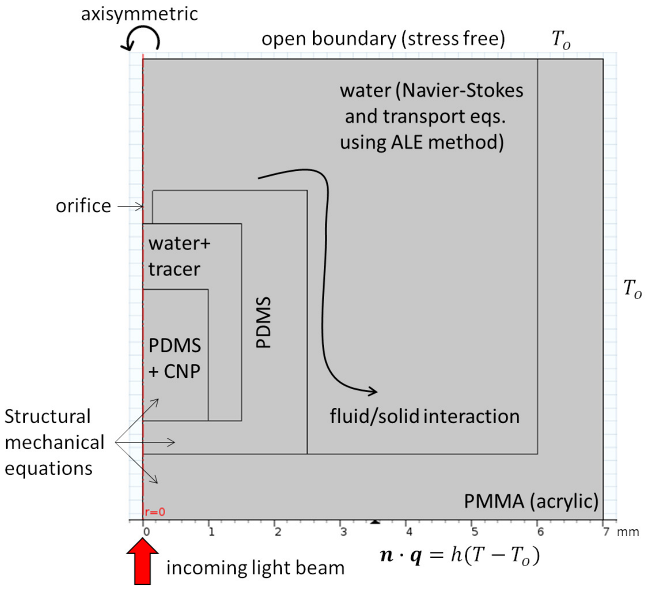

Figure 3: 2D axisymmetric domain and boundary conditions used in the fluid-structure interaction model.

In order to simulate the "squeeze" and liberation of the content by means of mechanical deformation, two additional governing equations were considered. First, the heat and mechanical equations have to be solved (transient formulation) in order to obtain the transient deformation of the elastic element (recall that the mechanical problem is formulated in the material coordinate frame ($\mathcal{X}$), while the spatial frame ($\mathbf{x}$) is not fixed and it is displaced according to $\mathbf{x} = \mathcal{X} + \mathbf{y}(\mathcal{X}, t)$). In addition, we also seek for the solutions for the fluid flow and transport of the marker solution, which are formulated in the spatial coordinate frame. Note also that the boundaries of the elastic elements can move and induce a flow in the liquid, and consequently transport of the marker solution. Because for the mechanical formulation the spatial frame is not fixed but it rather moves according to the material frame, the so-called Arbitrary Lagrangian Eulerian method (or ALE) can be used for solving the flow, liquid transport and the heat equations. The Navier-Stokes equations can thus be written as:

$$\begin{aligned} \rho\frac{\partial \mathbf{u}}{\partial t} + \rho[(\mathbf{u} - \mathbf{u}_{mesh}) \cdot \nabla]\mathbf{u} &= \nabla \cdot [-p\mathbf{I} + \mu(\nabla\mathbf{u} + (\nabla\mathbf{u})^T)] \\ \nabla \cdot \mathbf{u} &= 0 \end{aligned} \quad (9)$$

where p and μ are the pressure and viscosity of the liquid, respectively, while $\mathbf{u}_{mesh}$ is the velocity of the elastic boundaries (in the finite element method this is equal to the mesh velocity) prescribed by the mechanical problem. Due to the movement of the spatial reference frame, the time derivatives are computed as:

$$\frac{\partial \mathbf{u}}{\partial t} \approx \frac{\mathbf{u}(\mathbf{x}, t)^n - \mathbf{u}(\mathbf{x}, t)^{n-1}}{\Delta t}\ , \quad (10)$$

where n, $n-1$ denote not only the current and previous time, but also the current and previous physical frame. To track the content of the marker solution in the capsule, we also

included the diffusion-convective equation but without the diffusion term in order to consider only convection driven by the moving boundaries, i.e.:

$$\frac{\partial c}{\partial t} + \nabla \cdot [(\mathbf{u} - \mathbf{u}_{mesh})c] = 0 \ , \tag{11}$$

This is further complemented by the flux condition $j = c(\mathbf{u}_{mesh} \cdot \mathbf{n})$ at boundaries with normal vector $\mathbf{n}$ that moves due to the thermal expansion. For equation 9 we further impose the conditions $\mathbf{u} = d\mathbf{y}/dt$, $\sigma_{solid} \cdot \mathbf{n} = [-p\mathbf{I} + \mu(\nabla\mathbf{u} + (\nabla\mathbf{u})^T)] \cdot \mathbf{n}$ at the fluid/solid boundary, i.e., the stresses should be continuous at the water/PDMS boundary as well as the velocity field. Finally, for transient problems, the dynamic equation 4 now reads:

$$\rho\frac{\partial^2 \mathbf{y}}{\partial t^2} = \nabla \cdot \sigma_{ij} \ , \tag{12}$$

where σ_{ij} is computed at the current geometrical configuration, as done in the rest of the governing equations (the Cauchy stress tensor can be related to the second Piola-Kirchhoff stress tensor by well-known relations, see [2]). As initial condition, the whole domain is set to $T_o = 25°C$ and the temperature then starts to increase according to equation 2, along with the accompanying thermal expansion of the active PDMS/CNPs. In order to have a consistent initialization, the heat generation term is started with a smooth ramp with a time period of 55ms. Finally, it is important to mention that, in order to comply with mesh resolution constrains, the orifice is considered to be initially open. As shown in figure 3, such orifice is given initially by a free entrance of $310\mu m$ in diameter.

Displacement of the liquid tracer by air bubbles.

As mentioned in the experimental results, release of the marker solution from the capsule can also be driven by the isobaric expansion of air bubbles trapped inside the device. In a third simulation we tested numerically this mechanism as a possible way of liquid release. The full problem was approximated by two sequential studies: the first study yielded the average temperature evolution inside a bubble of 2.3mm of curvature radius (similar in size to those observed in the experiments); this was placed in a setup similar to that shown in figure 3 but only the heat transfer equation in a fixed spatial frame was solved. Using this "temperature history" we then computed the decrease of the air density as a function of time using the ideal gas law $\rho(t) = p \cdot M_w/\mathcal{R}T(t)$ for a constant pressure process (M_w is the molecular weight of air and $\mathcal{R}$ the ideal gas constant). In the second study we then solved the flow and transport equations, 9 and 11, using the ALE formulation (moving mesh) including the decrease of the air bubble density, $\rho(t)$. This should produce an expansion of the bubble by considering the more general mass conservation equation

$$\frac{\partial \rho}{\partial t} + \nabla \cdot (\rho\mathbf{u}) = 0 \ , \tag{13}$$

displacing the liquid inside the cavity and therefore inducing liquid release through the open hole. The gas/liquid interface was treated with the usual liquid/liquid boundary condition considering a surface tension of $72mN/m$. [4]

Effects of convective current.

In the fourth simulation we finally explored the possibility of liquid release by fluid flow induced by local density gradients in the liquid (water) due to the increase in temperature. In this problem we solved the heat, fluid flow and transport equations in steady state in a fixed geometry, similar to that depicted in figure 3 and considering a volumetric body force of $-\rho(T)\mathbf{g}$ in the Navier-Stokes equations, where $\rho(T)$ is the water density as a function of the temperature and $\mathbf{g}$ is the gravity vector. Figure 4 shows the results of this simulation and the flow patterns induced by such density gradients near the hole entrance. The main observation obtained from this simulation is that the flow currents inside and outside the cavity are not connected, but are rather separated forming independent vortex currents. This is further manifested by the fact that the marker solution, colored in red, remains inside the cavity once the flow currents are established due to the density gradients. We ran the simulation with different directions of the gravity vector but the results were similar: the marker solution always stays inside the cavity.

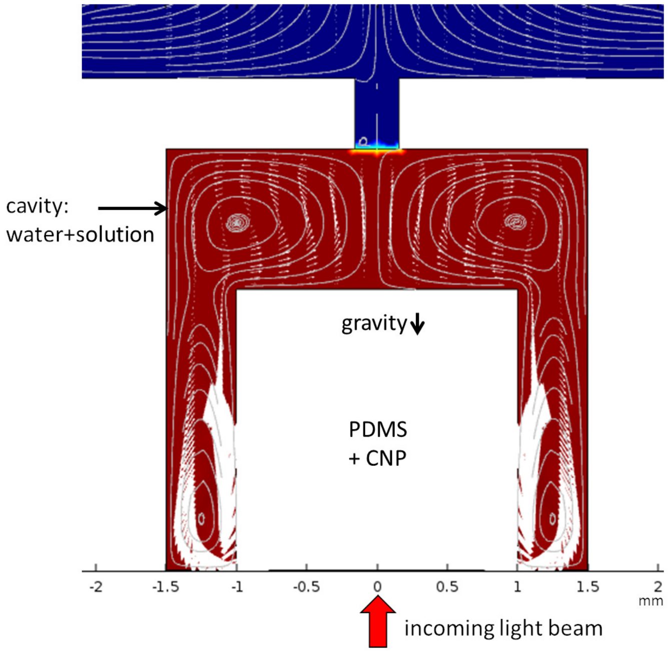

Figure 4: Flow field (white arrows) and streamlines (grey lines) produced by local density gradients due to the increase of temperature inside the capsule; $P_o = 179.1mW$, the color map denotes the concentration of a marker solution initially deposited inside the capsule ($10mol/m^3$).

	$\alpha[1/K]$	$C_p[J/(kg \cdot K)]$	$\rho[kg/m^3]$	$\kappa[W/(m \cdot K)]$	$E[MPa]$	ν	$\kappa_{ext}[mm^{-1}]$
PDMS	9.6×10^{-4}	1460	970	0.3	1.521	0.498	12.18
PMMA	5.1×10^{-5}	1570	1189	0.2	6129.9	0.32	
water		4187	1000	0.6			
air		1005	ideal gas law	0.026			

Table 1: Mechanical and thermal properties of the materials used in the simulations. PMMA stands for polymethyl-methacrylate or acrylic, the properties of the PDMS/CNPs are for the 1.0% composite formulation (in our simulations the properties of PDMS and PDMS/CNPs 1.0% were considered to be the same except that the value of κ_{ext} is zero in pristine PDMS). In COMSOL Multiphysics the properties listed here are considered functions of temperature and/or pressure; in the table we put typical values at $T = 25°C$.

1 References

[1] M. E. Gurtin, An Introduction to Continuum Mechanics, Academic Press (1981), USA New York.
[2] J. Bazilevs, K. Takizawa, T.E. Tezduyar, Computational Fluid-Structure Interaction, Methods and Applications, John Wiley and Sons (2013), UK.
[3] T.K. Kim, J.K. Kim, O.C. Jeong, Measurement of nonlinear mechanical properties of PDMS elastomer, Microelectron Eng. 88, 1982 (2011).
[4] A. Prosperetti, G. Tryggvason, Computational Methods for Multiphase Flow, Cambridge University Press (2009), UK.
